# Reliability of the active drag assessment using an isotonic resisted sprint protocol in human swimming

**DOI:** 10.1038/s41598-022-17415-5

**Published:** 2022-07-29

**Authors:** Tomohiro Gonjo, Bjørn Harald Olstad

**Affiliations:** 1 Department of Rehabilitation and Sport Sciences, Bournemouth University, Poole, UK; 2 Department of Physical Performance, Norwegian School of Sport Sciences, Ullevål Stadion, Postboks 4014, 0806 Oslo, Norway

**Keywords:** Fluid dynamics, Data acquisition, Scientific data

## Abstract

The purpose of the presents study was to investigate the reliability of the active drag (*D*_*a*_) assessment using the velocity perturbation method (VPM) with different external resisted forces. Eight male and eight female swimmers performed 25 m sprints with five isotonic loads (1–2–3–4–5 kg for females; 1–3–5–7–9 kg for males), which were repeated twice on different days. The mean velocity and semi-tethered force were computed for each condition, and the free-swimming maximum velocity was estimated with load-velocity profiling. From the obtained variables, *D*_*a*_ at the maximum free-swimming condition was calculated using VPM. Absolute and typical errors and the intra-class correlation (ICC) were calculated to assess test–retest reliability. 95% confidence interval (95% CI) lower bound of ICC was larger than 0.75 in 3, 4 (females only) and 5 kg trials in both sexes (corresponding to 37–60 N additional resistance; all p < 0.001), which also showed small absolute and relative typical errors (≤ 2.7 N and ≤ 4.4%). In both sexes, 1 kg load trial (16–17 N additional resistance) showed the lowest reliability (95% CI of ICC; − 0.25–0.83 in males and 0.07–0.94 in females). These results suggested that a tethered force of 37–60 N should be used to assess *D*_*a*_ using VPM.

## Introduction

In human aquatic locomotion, low hydrodynamic resistance from the water (active drag; *D*_*a*_) is often considered to be a key variable. However, due to the complex unsteady fluid phenomena, it is currently impossible to directly measure *D*_*a*_. Therefore, researchers have established indirect methods to estimate *D*_*a*_, which often require special devices. For example, di Prampero et al.^1^ measured swimmers’ oxygen consumption while swimming under assisted and resisted conditions in a circular swimming channel. They plotted the oxygen consumption against the external load on a two-dimensional plot, established a linear regression line on the plot, and mathematically estimated *D*_*a*_ by extrapolating the regression line to zero oxygen consumption. A similar mathematical method has also been developed in the last decades, such as the use of the residual thrust during swimming trials with different flow velocities while swimmers maintain their stroke frequency^2–4^. However, these methods require a swimming channel or flume, which is not accessible for many practitioners.

Another device that has been frequently used to assess *D*_*a*_ is the Measuring Active Drag (MAD) system, which requires the swimmer to propel by pushing off submerged pads equipped with force transducers^5^. Although the MAD-system has been widely used^6–8^, this method also requires sets of large pushing pads that are often not accessible to practitioners. Furthermore, the MAD-system enables researchers to estimate *D*_*a*_ only in the arm-only front crawl stroke, and investigating *D*_*a*_ in the four whole-body competitive swimming strokes (butterfly, backstroke, breaststroke and front crawl) with this system is not possible.

Currently, one of the simplest ways to assess *D*_*a*_ is the velocity perturbation method (VPM) proposed by Kolmogorov and Duplishcheva^9^, which only requires athletes to swim with their maximal effort with and without a known external resisted force via a non-elastic cord and an external load or object. *D*_*a*_ can then be mathematically computed using the equation below.

1 $${D}_{a}=\frac{{F}_{add}\cdot {v}_{2}\cdot {v}_{1}^{2}}{{v}_{1}^{3}-{v}_{2}^{3}},$$

where *F*_*add*_ is the additional resistive force due to the external load or object, and *v*_*1*_ and *v*_*2*_ are the velocities measured without and with the external load or object, respectively. Despite the simplicity, this method also has limitations, such as the assumption that the swimmer produces an equal amount of power during the free-swimming condition and the resisted swimming condition, which is questionable^10^. Furthermore, VPM assumes that *D*_*a*_ increases with the square of the swimming velocity; however, when the swimmer actively propels forward, this is not always the case^3,11^. Given these simplified assumptions, the accuracy of the data obtained by VPM might be questionable. However, despite the accuracy being not guaranteed, the method would be practically useful if it has strong reliability as the method can then be used to monitor the short-term and long-term changes in *D*_*a*_.

Researchers have applied this method using a wide range of additional resistances^12–14^. However, it is currently unknown how much force should be assigned to swimmers to ensure reliable outcomes. As violating the bespoken equal-power assumption systematically affects the outcome^10^, assigning a load or object that causes a small additional resisted force is probably preferable to make the two conditions as similar as possible. However, assigning too small resistance might cause a large random error because when the assigned resisted force is too small, a slight swimming motion (such as kicking and consequent splashes) might cause random movements of the cord (and the pulled object). This likely affects the swimmer’s velocity or the external force to which the swimmer is exposed. However, the effect of choosing different resisted forces in *D*_*a*_ calculation using VPM has not been assessed in the literature.

In summary, VPM is one of the most practical methods to quantify *D*_*a*_^15,16^ among many methods, but it is unknown how much additional force should be used for *D*_*a*_ calculation with this method. Furthermore, the reliability of the method has not been reported in the literature. Therefore, the purposes of the present study were to assess the reliability of VPM with different resisted forces and investigate the difference in the calculated outcomes between distinct conditions. It was hypothesised that small resistance conditions would result in low reliability in *D*_*a*_ calculation using VPM.

## Material and methods

### Participants

Eight males (17.0 ± 1.8 years age, 1.85 ± 0.05 m height, 73.0 ± 6.4 kg mass, 690.4 ± 67.8 FINA Points) and eight females (17.6 ± 1.2 years age, 1.71 ± 0.06 m height, 64.8 ± 7.2 kg mass, 689.6 ± 91.4 FINA Points) who specialised in front crawl were recruited.

### Procedures

A cross-sectional study design was used. The testing was performed in a 25 m indoor swimming pool (27 and 28 °C water and air temperature, respectively), where participants performed their individual warm-up procedure on land and in water as they usually do in competitions. Thereafter, swimmers were instructed to perform 5 × 25 m sprints with their maximum effort with five isotonic external loads (1, 2, 3, 4 and 5 kg for females and 1, 3, 5, 7 and 9 kg for males). Swimmers had at least 4 min of rest between each trial. The external load was assigned to the swimmer via a non-elastic cord using a portable robotic resistance device, 1080 Sprint (1080 Motion AB, Lidingö, Sweden), which also measured the swimming velocity and the tethered force (333 Hz sampling frequency). The device was positioned on the starting block resulting in the location of the origin of the cord exactly 1 m above the water surface. The cord was connected to the swimmer’s waist with an S11875BLTa swim belt (NZ Manufacturing, OH, United States), meaning that the measured velocity was the velocity of the abdomen region of the swimmer rather than their centre of mass. To investigate the reliability of *D*_*a*_ assessment, the same procedure was repeated twice at the same time on different days with a 1–5 days interval.

Three stroke cycles around the mid-pool were extracted using the time-velocity curve from the obtained data. The mean swimming velocity (*v*_*add*_) and tethered force (*F*_*add*_) during the three-cycle period at each condition were calculated. The horizontal component of the measured velocity and force were obtained using the equation below for the analysis using the trigonometric ratios^17–19^

2 $${var}_{H}=var\cdot cos\left[{sin}^{-1}\left(\frac{1.00}{{L}_{cord}}\right)\right],$$

where *var*_*H*_ and *var* are respectively the horizontal component and measured value of the variable, 1.00 is the height of the origin of the cord from the water surface, and *L*_*cord*_ is the length of the cord at the time. The maximum velocity (*v*_*max*_) at a free-swimming condition was estimated using the load-velocity profiling^17,18^, and *D*_*a*_ at *v*_*max*_ was computed using Eq. (1) with *v*_*max*_, *v*_*add*_ and *F*_*add*_ as inputs for each external load condition.

### Statistical analyses

The day-to-day reliability was assessed using intra-class correlation (ICC) with a two-way random single-measure model, and the absolute and percentage (relative to the mean) typical errors were quantified^20^. ICC was interpreted as showing a meaningful agreement when the 95% confidence interval (95% CI) lower bound was larger than 0.75^21^. A two-way repeated-measures analysis of variance (ANOVA) with Bonferonni correction for multiple post hoc comparisons was employed to investigate systematic day and external load effects. The normality of data was assessed using the Shapiro–Wilk test and confirmed for all *D*_*a*_ outcomes. ICC analysis and ANOVA were conducted using the Statistical Package for Social Sciences (SPSS) version 24.0 (IBM Corp, Armonk, NY, United States) with significance level of p = 0.05.

### Ethics approval and consent to participate

The study was approved by the local Ethical Committee and the National Data Protection Agency for Research in accordance with the Declaration of Helsinki. All participants (or a legal guardian for minors) were provided detailed verbal and written explanations of the purpose, procedure and risks related to the study and provided written informed consent.

## Results and discussion

**Figure 1:**
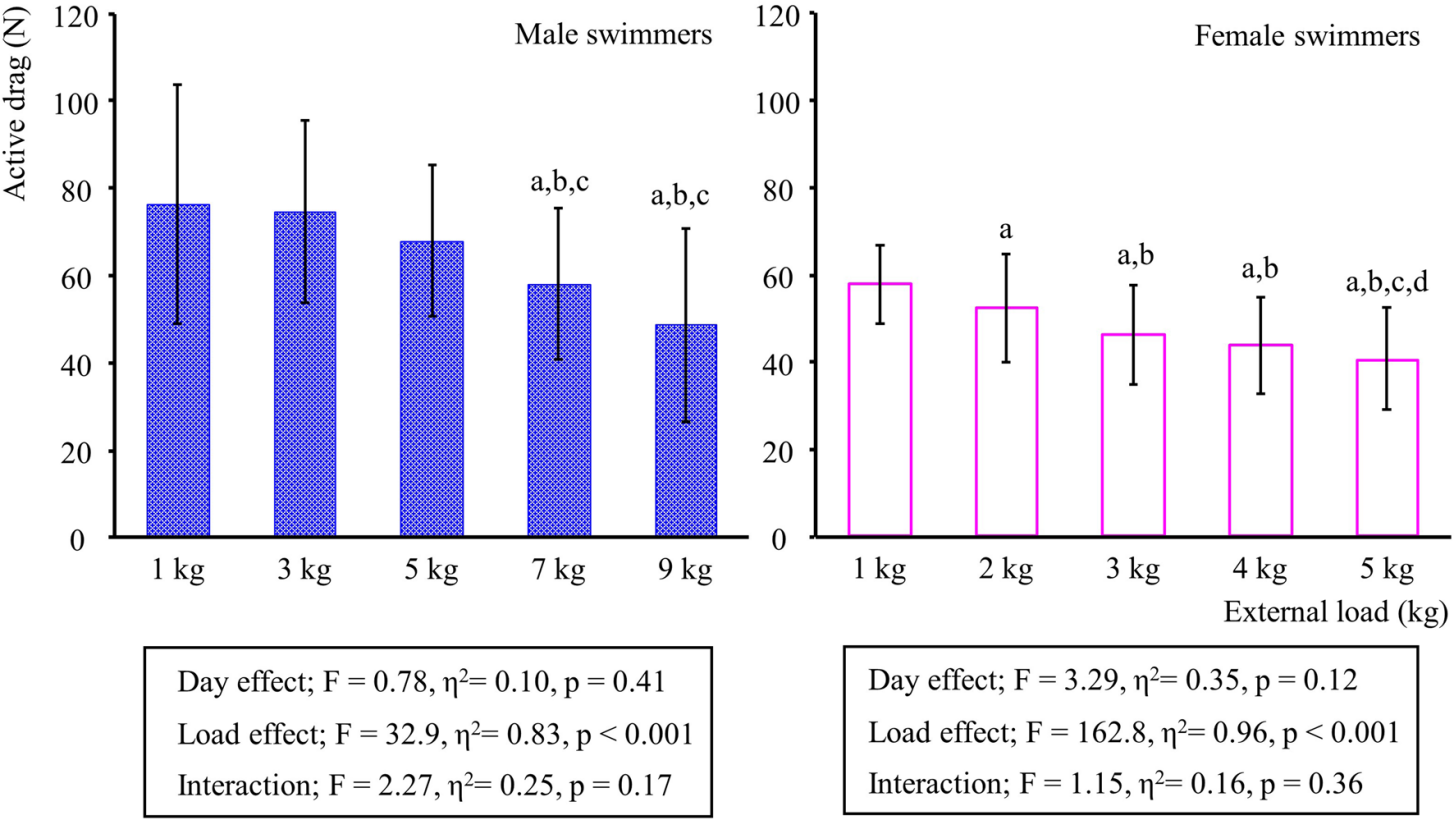
Active drag estimated from different external loads with results from a two-way repeated-measures ANOVA. Due to the non-significant day effect, the mean active drag between the two testing days is presented in the figure. Vertical bars are the standard deviation, and a, b, c and d show a significant difference from 1, 3, 5 and 7 kg (males) or 1, 2, 3, 4 kg trials (females), respectively.

Descriptive statistics and results from the reliability analyses are presented in Tables 1 and 2, respectively. In both sexes, trials with an external load smaller than 3 kg showed < 0.75 of the 95% CI lower bound. In male swimmers, this was also the case for 7 and 9 kg trials. The absolute and relative typical errors were similarly small at 3 and 5 kg trials in males and smallest at the 5 kg trial in female swimmers. In both sexes, a significant external load effect on *D*_*a*_ outcome was observed (p < 0.001), while neither a significant day effect nor the interaction between the effects was found (Fig. 1). In male swimmers, *D*_*a*_ measured with 7 and 9 kg external load were smaller than *D*_*a*_ obtained from 1, 3 and 5 kg trials. In females, all *D*_*a*_ values differed from those obtained in other trials, except for the comparison between 3 and 4 kg (p = 0.07).

**Table 1:** Mean (standard deviation) of the variables related to the active drag calculation.

	Male					Female				
	1 kg	3 kg	5 kg	7 kg	9 kg	1 kg	2 kg	3 kg	4 kg	5 kg
Day 1 *v*_*add*_ (m/s)	1.66 (0.09)	1.50 (0.14)	1.27 (0.21)	1.03 (0.22)	0.74 (0.31)	1.41 (0.09)	1.28 (0.10)	1.13 (0.12)	0.99 (0.13)	0.84 (0.16)
Day 2 *v*_*add*_ (m/s)	1.68 (0.09)	1.50 (0.14)	1.29 (0.15)	1.03 (0.23)	0.77 (0.30)	1.44 (0.08)	1.30 (0.10)	1.14 (0.10)	1.01 (0.13)	0.87 (0.15)
Day 1 *F*_*add*_ (N)	16.58 (0.17)	37.87 (0.26)	59.06 (0.37)	80.19 (0.42)	101.48 (0.43)	16.12 (0.21)	26.62 (0.27)	37.14 (0.12)	47.67 (0.13)	58.23 (0.34)
Day 2 *F*_*add*_ (N)	16.68 (0.16)	37.84 (0.25)	59.08 (0.28)	80.27 (0.35)	101.45 (0.38)	16.16 (0.15)	26.63 (0.18)	37.17 (0.15)	47.71 (0.18)	58.30 (0.22)
Day 1 *v*_*max*_ (m/s)	1.83 (0.06)					1.57 (0.10)				
Day 2 *v*_*max*_ (m/s)	1.82 (0.08)					1.56 (0.10)				
Day 1 Da at *v*_*max*_ (N)	83.50 (32.78)	73.33 (21.21)	67.29 (16.42)	57.73 (18.55)	50.80 (23.28)	59.44 (11.12)	52.64 (13.46)	45.73 (12.03)	44.26 (12.35)	40.95 (12.46)
Day 2 Da at *v*_*max*_ (N)	69.14 (20.50)	75.97 (22.09)	68.58 (19.41)	58.34 (17.38)	46.56 (22.25)	54.97 (6.40)	50.89 (12.31)	45.54 (11.68)	42.27 (10.38)	39.22 (11.77)

*v*_*add*_, mean swimming velocity with an additional resistance; *F*_*add*_, mean additional resistive force due to the external load; *v*_*max*_, estimated maximum velocity in a free-swimming condition; *D*_*a*_, active drag.

**Table 2:** Test–retest absolute typical error, typical error relative to the mean and the intra-class correlation coefficients obtained from the active drag calculation.

	Male					Female				
	1 kg	3 kg	5 kg	7 kg	9 kg	1 kg	2 kg	3 kg	4 kg	5 kg
Typical error (N)	17.65	2.68	2.60	3.54	3.68	3.98	2.39	2.01	1.56	1.17
Typical error (%)	23.12	3.59	3.83	6.10	7.56	6.96	4.62	4.41	3.60	2.93
ICC	0.40	0.94	0.95	0.92	0.91	0.71	0.88	0.97	0.97	0.96
ICC 95% CI_lower_	− 0.25	0.75	0.78	0.66	0.66	0.07	0.53	0.82	0.76	0.83
ICC 95% CI_upper_	0.83	0.99	0.99	0.98	0.98	0.94	0.97	0.99	0.99	0.99
ICC p-value	0.13	< 0.001	< 0.001	< 0.001	< 0.001	0.01	0.00	< 0.001	< 0.001	< 0.001

ICC, Intra-class correlation coefficient; 95% CI_lower_, lower bound of the 95% confidence interval; 95%CI_upper_, upper bound of the 95% confidence interval.

A non-significant day effect for both sexes showed that there was no systematic bias in the day-to-day reliability assessment. The low ICC observed in 1 kg (both males and females) and 2 kg (females), corresponding to *F*_*add*_ of 16–27 N (Table 1), suggests that low resistance should not be used to assess *D*_*a*_ with VPM and supports the initial hypothesis. Nevertheless, using too large resistance is also not advisable as male swimmers showed lower reliability when *D*_*a*_ was assessed with 7 and 9 kg (*F*_*add*_ = 80–102 N) compared with 3 and 5 kg load trials (*F*_*add*_ = 37–60 N). From the reliability perspective, researchers and practitioners should assess *D*_*a*_ with *F*_*add*_ of 37–60 N in both male and female swimmers.

The between-participants mean of *D*_*a*_ varied from 46 to 84 N in males and from 39 to 60 N in females (at the velocity of about 1.82 m/s and 1.56 m/s, respectively), depending on the external load assigned to the swimmer. Furthermore, the larger the external load used in VPM, the lower *D*_*a*_, as illustrated in Fig. 1. Even though it is not possible to discuss the accuracy of the method as there is currently no method that directly measures *D*_*a*_, comparing the results from the current study with the literature is helpful to examine whether the obtained *D*_*a*_ in the present study is reasonably aligned with previous studies. Kolmogorov and Duplishcheva^9^ analysed *D*_*a*_ for whole-body front crawl swimming in both males and females and reported that, on average, males showed about 83 N at 1.78 m/s and females exhibited about 53 N at 1.60 m/s. These values were similar to the results of the present study (with 1 kg external load). Another study^22^ investigated *D*_*a*_ in arms-only front crawl swimming using MAD-system and established *D*_*a*_ equation for males (*D*_*a*_ = 28.9·*v*^2.12^) and females (*D*_*a*_ = 20.4·*v*^2.28^). These equations, in combination with the mean *v*_*max*_ in the present study, generate *D*_*a*_ = 100.5 N for males and *D*_*a*_ = 56.2 N for females. This *D*_*a*_ for females is comparable to the result of the present study (when the resisted load was 1 kg). However, the *D*_*a*_ calculation for males produced a slightly larger value than the present study, which might be due to the previous study having included water polo players in their samples^22^. However, a recent study^23^ assessed *D*_*a*_ for both males and females using the assisted towing method and reported considerably larger *D*_*a*_ than other studies, including the present one (mean *D*_*a*_ = 89.0 N for females at *v* = 1.60 m/s, and mean *D*_*a*_ = 140.5 N for males at *v* = 1.87 m/s).

The comparisons between the previous studies and the present study showed that the results of the present study were the closest to the literature when the external load for VPM was 1 kg. Furthermore, *D*_*a*_ calculated in heavy load conditions (such as 5–9 kg loads for males and 3–5 kg loads for females) were close to, or even smaller than, passive drag results reported in the literature. For example, Zamparo et al.^24^ showed the passive drag of 70 N at 1.80 m/s and 47–60 N at 1.42–1.62 m/s for male and female competitive swimmers, respectively. These passive drag values are larger than *D*_*a*_ found in the present study with 5–9 kg (males) and 3–5 kg loads (females), which indirectly suggests that the *D*_*a*_ obtained at heavy load conditions were probably underestimated.

These examples imply that there is likely a trade-off between the accuracy and the reliability of VPM, i.e. the lighter the external load, the more accurate but less reliable the *D*_*a*_ outcome. As indicated in the introduction, VPM is very sensitive to the violation of its assumption that the swimmer’s power output is equal between without and with external force/load conditions^10^. As measuring the power output during swimming is currently a very challenging task, it is unclear how much the external force/load affected the power output of swimmers. However, assuming that the power output is more similar when the two conditions (with and without external resistance) are closer, it is reasonable to consider that the power output in a semi-tethered condition is closer to free-swimming when assigning a smaller force/load.

Therefore, it is necessary to choose the external load which can produce reliable results while avoiding assigning a heavy load to the swimmer. For male swimmers, considering that 3 kg and 5 kg trials showed high reliability and there were no statistical differences in *D*_*a*_ between these trials, *D*_*a*_ assessment with *F*_*add*_ of 37–60 N can be equally recommended. In females, among the three trials that exhibited high reliability (3–4–5 kg), 5 kg load produced a significantly lower *D*_*a*_ than 3 kg and 4 kg trials, meaning that the underestimation of *D*_*a*_ was probably more severe in the 5 kg trial than in the other two trials. Therefore, even though assessing *D*_*a*_ with *F*_*add*_ of 37–60 N could produce reliable results, limiting *F*_*add*_ to 37–47 N might be preferable to minimise the underestimation of *D*_*a*_ for females.

In conclusion, VPM can produce reliable results when assigning swimmers with a 3–5 kg load (37–60 N *F*_*add*_) for both male and female competitive swimmers, and assigning smaller or larger *F*_*add*_ than the suggested range to swimmers can cause low measurement reliability. The calculated *D*_*a*_ outcomes with this range of *F*_*add*_ are likely underestimated. Nevertheless, due to strong reliability, VPM with *F*_*add*_ of 37–60 N can be used to assess differences in *D*_*a*_ between groups or to assess a long-term change in *D*_*a*_, as long as the same setting is utilised. However, it is advisable to limit *F*_*add*_ to 37–47 N for females due to the underestimation of *D*_*a*_ being more severe when assigning a larger *F*_*add*_, such as 60 N. The present study only focused on post-puberty age swimmers, but VPM has also often been used to assess *D*_*a*_ in young swimmers^16^. Therefore, the reliability of this method for age group swimmers should be further investigated.

## Data Availability

The datasets used and/or analysed during the current study are available from the authors on reasonable request.

## Abbreviations

*D*_*a*_: Active drag
VPM: The velocity perturbation method
*F*_*add*_: Additional external force added to swimmers in a semi-tethered swimming trial
*v*_*add*_: The mean forward swimming velocity during a semi-tethered swimming trial
*v*_*max*_: The mean swimming velocity during a maximum free-swimming condition estimated from the load-velocity profiling
ICC: Intra-class correlation
95% CI: The 95% confidence interval
ANOVA: Analysis of variance
